# Phenolic Composition Influences the Effectiveness of Fining Agents in Vegan-Friendly Red Wine Production

**DOI:** 10.3390/molecules25010120

**Published:** 2019-12-28

**Authors:** Susana Río Segade, Maria Alessandra Paissoni, Mar Vilanova, Vincenzo Gerbi, Luca Rolle, Simone Giacosa

**Affiliations:** 1 Dipartimento di Scienze Agrarie, Università degli Studi di Torino, Forestali e Alimentari, Largo Paolo Braccini 2, 10095 Grugliasco (TO), Italy; susana.riosegade@unito.it (S.R.S.); mariaalessandra.paissoni@unito.it (M.A.P.); vincenzo.gerbi@unito.it (V.G.); simone.giacosa@unito.it (S.G.); 2 Misión Biológica de Galicia (CSIC), El Palacio-Salcedo, 36143 Pontevedra, Spain; mvilanova@mbg.csic.es

**Keywords:** fining agents, vegetal proteins, phenolic compounds, astringency, color, red wine

## Abstract

Plant proteins have been proposed as an alternative to animal-origin proteins in the wine industry because they are allergen-free and vegan-friendly. The aim of this study was to evaluate the effectiveness of plant proteins as fining agents on red wines with different phenolic composition. Two formulations for commercially available vegetal proteins (potato and pea origin) were assessed at two doses to modulate the fining treatment to the wine phenolic profile. The results evidenced that fining agents derived from plants have different levels of effectiveness on the removal of phenolic compounds depending on the origin, the formulation used, dose applied, and also wine characteristics. On Nebbiolo wine, the study was particularly significant due to its phenolic composition. One pea-based fining agent had an effect comparable to gelatin (animal origin) on the removal of polymeric flavanols with a minor loss of anthocyanins and therefore better preserving the wine color in terms of intensity and hue. For Primitivo, Montepulciano, and Syrah wines, even though there was a formulation-dependent effect, vegetal proteins gave more balanced reductions in terms of target phenolic compounds contributing to astringency and color perception.

## 1. Introduction

The use of fining agents in winemaking is widely known as a processing aid to clarify, enhance the wine stability, remove off-flavors, and, particularly in red wines, soften sensory properties such as bitterness and astringency by modulating phenolic composition [1]. Traditional fining agents based on proteins of animal origin, including casein, egg albumin, gelatin, and isinglass, are commonly used to remove protein-reactive phenolic compounds [2]. However, color losses and unpleasant aromas can sometimes occur in the wine after the fining treatment [3]. From the health point of view, animal proteins have allergenic or intolerant potential and their residual presence in the wine may pose an important risk in sensitive individuals [1,4]. In this sense, according to EU regulation No. 579/2012 [5], all potentially allergenic fining agents present in the wine at a concentration higher than 0.25 mg/L have to be declared on the wine label. In the last recent years, cultural and ideological aspects are also increasingly influencing the wine market. Thereby, the global increase in vegetarian and vegan consumers has highlighted the necessity to make wines using fining products that represent a possible alternative to animal-derived fining agents. For this purpose, vegetal proteins have been already isolated from cereals, legumes, potatoes [6,7], seaweeds, grape seed extracts [8,9], and yeasts [10,11]. Other vegetal non-proteinaceous products, such as polysaccharide-based agents isolated from cell wall material of fresh apples and grapes or their respective pomaces, have shown great potential for wine fining purposes [12,13,14,15].

Focusing on protein-based fining agents, they can have a different affinity to different types of phenolic compounds. Proteins interact with wine phenolic compounds initially through hydrogen bonding and hydrophobic interactions, forming soluble complexes. In fact, gelatin is a highly efficient fining agent because of its potential hydrogen binding sites [3]. Subsequently, phenolic compounds are removed by precipitation through self-association of the complexes formed [16] or formation of insoluble protein aggregates (cross-linkages between proteins) incorporating the target species [17]. This precipitation induces reduced astringency and depends on the protein to tannin ratio as well as on the amino acid composition, tertiary structure, hydrophobicity, and molecular mass of proteins [2,18]. It is well-known that proline-rich proteins have a strong affinity to phenolic compounds and therefore they are of great interest for wine fining [19]. Concretely, aromatic ring structures of phenolic compounds have hydrophobic properties and the presence of galloylated units enhances the hydrophobic interactions with proline-rich proteins [20].

Among allergen-free vegetal proteins isolated from cereals, corn zeins are a heterogeneous protein mixture made of polypeptides differing in molecular mass, aggregation state, and isoelectric point. The effectiveness of zein as a fining agent could be due to their relative hydrophobicity, which is associated with the high content of non-polar amino acids [21,22]. In Cabernet Sauvignon, Merlot, and Valpolicella red wines, these authors reported that corn protein doses ranging from 5 to 15 g/hL are able to decrease wine turbidity and remove phenolic compounds, such as anthocyanins (−2.90–11.64%) and proanthocyanidins (−5.40–19.26%) after settling for 48 h at 20 °C, in a similar way to animal gelatin while preserving red wine color. Higher variability is found in the scientific literature in wine fining effectiveness for rice-based proteins. They have relatively low proline content and therefore only reduce slightly total tannins and astringency, showing also a low influence on the Cabernet Sauvignon wine color after settling for 48 h at 20 °C [7]. However, the use of molecular mass fractions ranging from 10 to 32 kDa promotes the removal of phenolic compounds that contribute to bitter and astringent perception and, at the same time, preserves the color of Samtrot and Lemberger red wines after storing in the dark at 8 °C for 24 h [23].

Grape-endogenous proteins sourced from grape seed extracts (GSE) have also shown good performance in decreasing Chardonnay white wine turbidity and increasing oxidative stability. In the Raboso red wine, a reduction in some anthocyanins (−1.0–4.0%) and proanthocyanidins (−3.2–5.9%) was observed, similarly to other animal- and vegetal-derived fining agents tested, without markedly affecting wine chromatic characteristics (less than −5%). Nevertheless, the most important impact of GSE corresponds to the sensory attributes, reducing the vegetal notes in the Raboso rosé wine and both acidity and astringency in the Raboso red wine [8]. The dosages used were 5, 10, and 20 g/hL of GSE, corresponding respectively to 2.2, 4.4, and 8.7 g/hL of protein.

Despite the effort made by various research groups in relation to the use of vegetal proteins as wine fining agents, few have been commercialized until now. Some of these products are based on Patatin P. It is a major 39–45 kDa glycoprotein from potato, with low proline content, whose fining ability has been demonstrated in red wines. Different studies have evidenced that proteins from potato reduce total tannin content and astringency similarly to gelatin, and even better than other fining agents in Aglianico and Cabernet Sauvignon red wines [6,7]. Nevertheless, these two studies reported contrasting results in relation to the wine color. The use of patatin for wine fining during one week at 14 °C preserves the chromatic characteristics of red wine, even though it removes 30% of monomeric anthocyanins at 30 g/hL dose in Aglianico red wine [6], whereas a significant decrease in the hue parameter is found in Cabernet Sauvignon after settling for 48 h at 20 °C [7]. Therefore, the wine phenolic composition and the fining conditions (dose, temperature, and time) may influence the impact of fining agents. In addition, other studies highlighted that potato protein with molecular mass fractions between 10 and 32 kDa reduces efficiently non-polymeric bitter and astringent marker compounds in red wines, such as caftaric acid, (+)-catechin, and (−)-epicatechin, with a minor impact on color intensity [23].

Other commercialized products are legume-derived proteins extracted from pea, although lentil, soybean, and lupine have been also studied as possible sources of vegetal protein. All of them are efficient fining agents when compared to gelatin, but soybean and lupine are considered allergenic substances [1]. In Aglianico red wine, either young or aged for twelve and twenty-four months, a recent study has reported that pea protein shows limited interaction with proanthocyanidins after settling for 168 h at 20 °C [18]. However, selective enzymatic hydrolysis, reducing the protein size but keeping hydrophobic binding sites intact, could enhance the accessibility to phenolic compounds and therefore improve the fining efficiency of pea protein. Lentil and soybean proteins, followed by hydrolyzed pea protein, remove efficiently proanthocyanidins in red wines (about 30–40% for monomers and 70–80% for proanthocyanidin dimers and trimers). An important advantage of pea protein isolate is the preservation of anthocyanins, whose losses occur and are particularly noticeable in young red wine so color tonality is not influenced by wine fining with pea, lentil, soybean, or gelatin proteins [18]. Other authors confirmed the lower efficiency of pea proteins to remove tannins when compared to gelatin and potato proteins [7], but they evidenced that the three vegetal-derived proteins reduce similarly the astringency of Cabernet Sauvignon wine. Similarly, the pea protein had a minor impact on proanthocyanidins in Catalanesca white wine [24]. In white wine, another study reported that pea protein could be an alternative to potassium caseinate by decreasing similarly total phenols (about −9%) and wine color (about −11% to –12%) at 0.4 g/L dose, being pea protein less efficient at reducing the browning potential [25].

There are few commercialized products alternative to the use of animal allergenic proteins for wine fining and some studies have provided contradictory results. Considering the increasing demand for the production of wines suitable for allergen-sensitized, vegetarian, and vegan consumers, the study aimed to compare the effectiveness of commercialized vegetable proteins derived from pea and potato, and the traditionally used fining agent (i.e., animal gelatin) at two different doses. Concretely, their ability to both remove monomeric, oligomeric, and polymeric flavanols that are involved in the wine astringency perception and preserve anthocyanins and color characteristics was assessed. The study was carried out on four monovarietal red wines, characterized by different phenolic content and composition, to assess the possibility of adapting the fining agent and dosage to a specific type of wine and/or phenolic profile. To our knowledge, no scientific study has been published on vegetal proteins derived from pea and potato for the fining of wines with different phenolic composition.

## 2. Results and Discussion

The wines were selected on the basis of the different phenolic composition, particularly the content of oligomeric and polymeric flavanols (FRV and PRO, respectively), as well as the content of anthocyanins (TA) (control wines, Table 1 and Table 2). The lower FRV/PRO ratio corresponds to Montepulciano wine (0.38), followed by Primitivo (0.41), and this ratio increases in Syrah and Nebbiolo wines (0.54 and 0.59, respectively). Regarding anthocyanins, Nebbiolo wine is characterized by a low content (112 mg/L) whereas Syrah is the richest wine in these red pigments (367 mg/L), followed by Montepulciano and Primitivo (275 and 255 mg/L, respectively).

### 2.1. Total Phenolic Compounds and Flavanols

Regarding Primitivo wine, total phenolic compounds evaluated through the A_280_ parameter only showed a significant difference for the high dose of GE (gelatin, conventional treatment) with respect to untreated wine (−8.6%, *p* < 0.001; Table 1). By contrast, using more comprehensive parameters such as the content of polymeric and oligomeric flavanols (PRO and FRV, respectively), a significant decrease was found for several fining treatments when compared to control wine. PRO content was significantly reduced when the wine was treated with gelatin independently on the dose (−10.3% and −13.9%, *p* < 0.001 for low and high dose, respectively), as occurred with the PT1 treatment (potato-based) at low dose and PE1 (pea-based) at the two doses studied (−7.8% and −8.8%, *p* < 0.001 for PT1L and PE1H, respectively). PE1L treatment reduced both PRO and FRV contents (−7.1%, *p* = 0.004 and −11.1%, *p* < 0.001, respectively), and it was the treatment that most significantly affected FRV. This decrease could be exploited in wine production because low molecular fractions of flavanols contribute greatly to wine bitterness [26].

In Montepulciano wine, even if no significant differences in total phenolic compounds (assessed by A_280_) occurred using different fining agents, variations in the flavanol content were found for both FRV and PRO values (Table 1). GE treatments significantly reduced both parameters with respect to the control (−12.1% and −18.9%, *p* < 0.001 for PRO, and −7.1%, *p* = 0.006 and −17.9%, *p* < 0.001 for FRV, for low and high dose, respectively), highlighting their effectiveness. The only vegetal fining agent that allowed a decrease of both PRO and FRV contents was PT1, giving a significant removal of PRO (−11.6%, *p* < 0.001 and −9.5%, *p* = 0.006 for low and high dose, respectively) and FRV (−11.2% and −12.8%, *p* < 0.001 for low and high dose, respectively). Instead, PE1 treatments resulted to be effective mainly on the reduction of low molecular flavanol fraction (FRV, −7.0%, *p* = 0.006 and −10.8%, *p* < 0.001 for low and high dose, respectively) while PRO contents showed a non-significant decrease when compared to control wine.

In the case of Syrah wine, total phenolic compounds (A_280_) determination after wine fining unveiled a significant decrease only using the high dose of gelatin with respect to the control (GEH, −8.0%, *p* = 0.002; Table 1). However, the more detailed flavanol estimation using PRO and FRV parameters evidenced, for GE and PT2 at high dose, a decrease of both PRO (−12.6%, *p* < 0.001 and −6.7%, *p* = 0.004, respectively) and FRV (−9.9%, *p* < 0.001 and −9.2%, *p* = 0.003, respectively). In addition, PE2 and PT1 at high dose, as well as PE1 at a low dose, had a significant impact only on PRO contents (−11.7%, −15.3%, and −7.4%, respectively, *p* < 0.01), the second fining condition (PT1H) presenting the highest PRO decrease observed among treatments. This aspect is of particular relevance because wine astringency is primarily driven by polymeric flavanols [26]. Moreover, the FRV parameter showed a significant decrease (−10.0%, *p* < 0.001) also after PT1 treatment at a low dose when compared to the control wine.

The main differences found in this study concerned Nebbiolo wine because it was strongly affected by the different fining treatments in terms of A_280_, PRO, and FRV (all *p* < 0.001; Table 1). The unusual phenolic profile of this wine, which is characterized by a limited anthocyanin content and a typically high flavanols content with respect to the other varieties evaluated, undoubtedly played a major role in this outcome. A significant reduction of A_280_ was observed after all treatments (from −3.3% up to −8.6%, *p* < 0.01). In fact, this decisive reduction of A_280_ could be justified by the removal of oligomeric and polymeric flavanols (FRV and PRO, respectively). The higher decrease in A_280_ with respect to the control wine was found for GE treatment at both low and high doses and for PT1 at high dose (−7.5%, −8.6%, and −7.4%, *p* < 0.001 for GEL, GEH, and PT1H, respectively). In accordance with the A_280_ parameter, PRO content was significantly reduced by all treatments with respect to the control (from −5.8% up to −15.4%, *p* < 0.01) with the exception of PT2 at both low and high doses. In particular, GEH was the most effective fining agent in the removal of both PRO and FRV with respect to the control (−15.4% and −13.8%, respectively, *p* < 0.001). As well, also PE1 reduced these two parameters (−9.6% and −9.7%, *p* < 0.001 for PRO, −7.4% and −10.4%, *p* < 0.001 for FRV, for low and high dose, respectively) as also PT1 at low dose did (−8.5% and −7.5%, *p* < 0.001 for PRO and FRV, respectively). By contrast, PE2, GEL, and PT1H treatments led to a PRO decrease (*p* < 0.01) without significantly affecting the low molecular flavanol component (FRV).

In the present study, the removal of flavanols during wine fining was similar to or even higher than that previously published for the use of gelatin, potato-based proteins (about −9% for PRO using both patatin and gelatin at 10 and 30 g/hL dose, FRV ranging from −1.1% to −13.9% for patatin and from −7.1% to −18.8% for gelatin at 10 and 30 g/hL [6]), and pea-derived proteins (−6.8% for PRO at 10 g/hL dose [8]; −3–5% of catechin and epicatechin and −15–22% of oligomeric proanthocyanidins at 20 g/hL dose [18]). The effectiveness of GEH fining agent to remove oligomeric and polymeric flavanols is undoubted as this treatment reduced PRO and FRV contents more than vegetal-derived proteins in Montepulciano and Nebbiolo wines, which are richer in polymeric proanthocyanidins, and was highly effective in Primitivo and Syrah wines. However, this study highlighted for the first time that both the effectiveness of vegetal-derived proteins and the target compounds are influenced by the wine phenolic composition. Specifically, the PE1L treatment reduced FRV content more efficiently than gelatin in Primitivo wine whereas PT1H removed a significantly higher PRO content than gelatin in Syrah wine. In Montepulciano and Syrah wines, which are characterized by the greatest richness in total phenolic compounds (expressed as A_280_, Table 1), PT1H treatment showed a decrease in low molecular flavanols similar to GEH. Instead, the same PT1H treatment allowed to reduce the PRO content in Syrah and Nebbiolo wines having a high FRV/PRO ratio. A decrease in FRV content not significantly different from that obtained for GE1H was achieved using PT1L and PE1H in Syrah and Nebbiolo wine, respectively.

Fining trials showed that polymeric proanthocyanidins are reduced significantly by a higher number of vegetal fining agents with respect to low molecular mass flavanols, probably due to they are firstly precipitated. The higher number of phenolic rings present in the more polymerized proanthocyanidins increases their hydrophobicity and therefore facilitates flavanol-protein interactions [17]. In addition to polymerization degree, other characteristics of flavanols such as galloylation percentage and conformational flexibility may play an important role in removing these phenolic compounds. In fact, galloylated proanthocyanidins are removed in a preferential way [17]. Moreover, hydrophobic interactions could be mainly responsible for precipitation by vegetal proteins derived from pea and potato, and some studies have confirmed the relatively high surface hydrophobicity of patatin [17,27].

### 2.2. Total Anthocyanins and Color Parameters

A side effect of wine fining is the removal of colored pigments, which may lead to a wine with reduced color quality. Different results were obtained depending on the fining agent used, the dose applied, and the variety in terms of total anthocyanins (TA), color intensity (CI), and tonality (hue) as reported in Table 2.

In Primitivo wine, significant differences between the wines treated with different fining agents and the untreated wines (control) were found for TA and CI parameters whereas the hue of the treated wines agreed with that of the control wine. TA content was significantly decreased with respect to the control sample when the wine was treated with gelatin at both low and high doses (−6.8%, *p* = 0.004 and −7.0%, *p* = 0.037 for GEL and GEH, respectively). As well, the high dose of PE1 (pea-based) reduced significantly anthocyanins, leading to a decrease of −7.7% for PE1H (*p* = 0.014). In the case of gelatin, also a CI decrease was reported with respect to the control (−6.8% and −12.8% for GEL and GEH, respectively, both *p* < 0.001), in agreement with the anthocyanins loss. In contrast, even if there was no significant reduction of TA content, PT1 treatment at a high dose led to a decrease of CI value with respect to the control (−9.5%, *p* < 0.001 for PT1H). Although the anthocyanin content mainly determines wine color, the CI parameter can be magnified by the presence of co-pigments, such as flavonols and flavanols, which may have been removed by the protein-based treatment. In general, a similar effect of fining treatment was observed on the three color components (yellow, red, and blue as A_420_, A_520_, and A_620_, respectively). However, GEL, GEH, and PT1H samples evidenced a higher decrease in the blue color component (A_620_, data not shown), which is related to co-pigmentation effects [28].

As found for Primitivo, lowered TA content was evidenced in Montepulciano treated wines depending on the fining treatments, whereas CI value was significantly reduced in all cases with the exception of PE2 at a low dose (from −2.7% up to −11.7%, *p* < 0.01) when compared to untreated wine. GEH and PT1H treated wines reported the lowest TA content, with a loss of −6.6% and −6.2%, respectively, compared to the control wine (both *p* < 0.001), followed by GEL and PE2H treatments with a loss of −3.7% and −3.5% (both *p* < 0.001) in TA content, respectively. This anthocyanin loss resulted in an increase in the hue parameter for PT1H treatment (+1.0%, *p* < 0.001). Even if to a lesser extent, also PT1L and PE1H showed lower TA content with respect to the control wine (both −2.8%, *p* = 0.002). In Montepulciano, a significant loss of anthocyanins resulted in lower color intensity. Anyway, the CI value was also reduced when TA content was not affected. The removal of oligomeric flavanols can support this reduction in PE1L sample (Table 1), however, the blue color component was more strongly affected than yellow and red components in wines treated with PE1L, PT2L, and PT2H, evidencing that the removal of other co-pigments, such as flavonols, may have occurred.

In Syrah wine, there was no significant loss of anthocyanins, but CI and hue were changed significantly for several fining treatments (both *p* < 0.001). In particular, CI value was reduced in all treated wines, with the exception of PT2L treatment, decreasing from −3.1% up to −9.0% (all *p* < 0.01) with respect to the control samples. The highest color losses corresponded to GEH and PT1H treatments (−9.0%, *p* < 0.001), followed by PT1L (−5.8%, *p* < 0.001) and GEL (−4.7%, *p* < 0.001). Interestingly, hue value was often lower in treated samples (decreasing from −0.6% to −1.0%, *p* < 0.01; except for PE2L, PT1L, and PT2H), meaning a lower yellow color component (A_420_) with respect to the red component (A_520_) and, therefore, the wine shifted to a red hue instead of yellow one. Considering that no change affected monomeric anthocyanins (data not shown), it is possible to hypothesize a decrease in anthocyanin-derived pigments, such as pyranoanthocyanins, which contribute to the orange hue of wines [29]. Granato et al. [18] reported losses of individual anthocyanin-derived pigments in Aglianico red wine treated with plant-based proteins and evidenced that gelatin removed monomeric anthocyanins and anthocyanin-flavanol adducts whereas pea-based proteins were ineffective.

Nebbiolo wine was strongly influenced by all fining treatments: a significant reduction from −8.0% to −21.5% (*p* < 0.01) of TA was found with respect to the control sample. The higher anthocyanin loss was reported for PE2 treatment at high dose, followed by gelatin at both the low and high doses, and PT1 at high dose (−21.5% for PE2H, −18.1% for GEL, GEH, and PT1H, all *p* < 0.001). This loss was reflected also in a significant decrease of CI value in all treated samples with respect to the control (from −3.9% to −15.6%, *p* < 0.05), which was particularly evident for GE treatment at both low and high dose, and PT1 at high dose (−10.1%, −15.6%, and −10.7% for GEL, GEH, and PT1H, respectively, all *p* < 0.001). Nevertheless, the highest loss of anthocyanins is not in correspondence with the highest decrease in color intensity. Despite the differences in CI and TA parameters, the hue was significantly increased only in PT1H wine with respect to the control (+1.3%, *p* = 0.011). In Nebbiolo, as well as in Montepulciano, the increased hue, in contrast with Syrah treatments’ effect, could be justified by the TA decrease, leading to a loss of red color component (A_520_), which was less observed in Syrah. This behavior in Syrah could be linked to the greatest richness in anthocyanins and to an anthocyanin profile prevalent in malvidin-3-glucoside with a high ratio of acylated derivatives, giving high anthocyanin stability [30]. In this aspect, Nebbiolo wines are particularly different because of their lower anthocyanin content and less abundance of acylated derivatives [31].

Anthocyanin losses during wine fining have been also reported in many studies, but their magnitude is variable. In Aglianico red wine, Gambuti et al. [6] reported a removal of total anthocyanins lower than −4% for patatin and gelatin at doses between 10 and 30 g/hL, whereas Granato et al. [18] evidenced losses of about −31% for gelatin and of −4% for pea proteins at doses of 20 g/hL. According to these studies, gelatin (animal origin) and potato-based vegetal proteins reduced more significantly the content of petunidin-, peonidin-, and malvidin-3-glucoside [6] whereas cyanidin-3-glucoside was negatively influenced by pea-derived proteins [18]. In the present work, the results obtained are in agreement with these findings. In fact, the anthocyanin losses in Primitivo and Nebbiolo wines, which are richer in cyanidin-3-glucoside than Montepulciano and Syrah, were higher after fining treatments with pea-derived proteins (PE1H and PE2H, respectively) whereas the lowest contents of anthocyanins corresponded to both GEH and PT1H treatments in Montepulciano and Syrah wines.

### 2.3. Comparison between Flavanol Reduction and Color Modification

**Figure 1:**
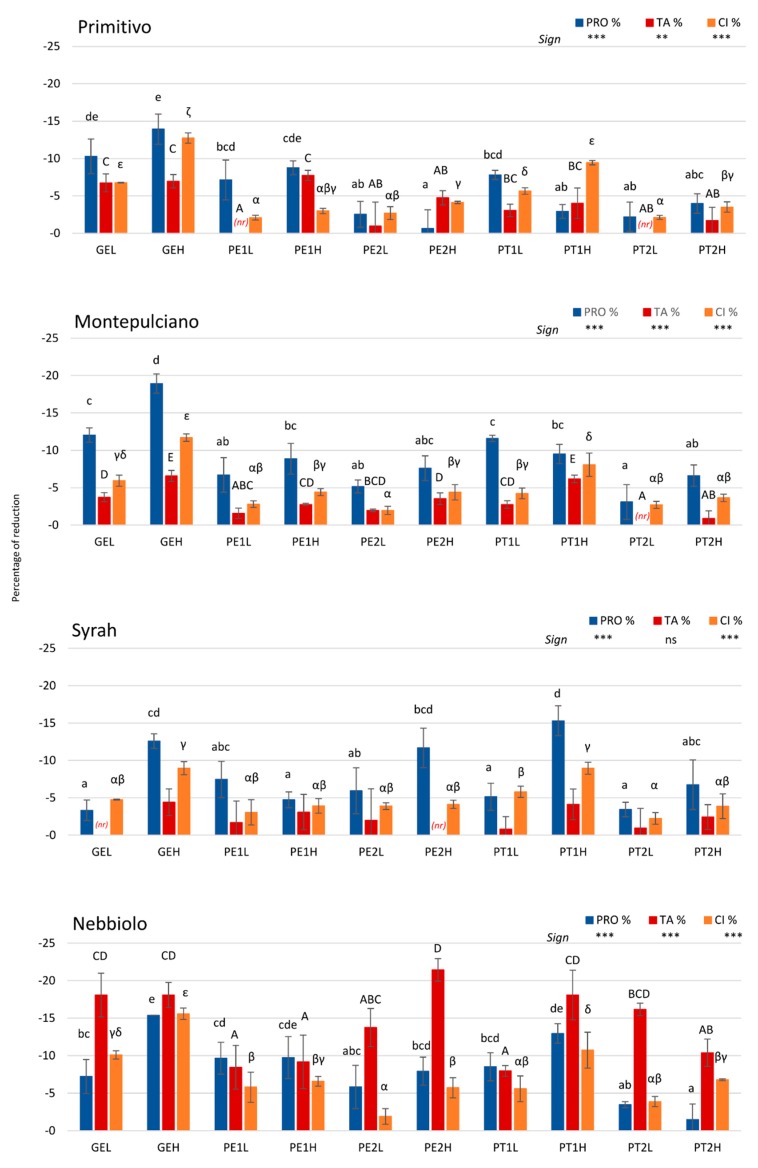
Reduction percentage of wine proanthocyanidins (PRO) after fining treatments with proteins of animal origin like gelatin (GE) and of vegetal origin from pea (PE1, PE2) and potato (PT1, PT2) with respect to the untreated wine, compared to reduction percentage of total anthocyanins (TA) and color intensity (CI). All data are expressed as the average value of individual treatments with respect to the control ± standard deviation (*n* = 3). Different lowercase Latin letters for PRO, different uppercase Latin letters for TA, and different lowercase Greek letters for CI indicate significant differences among treatments according to Tukey test (*p* < 0.05), respectively. Sign: **, ***, and ns indicate significance at *p* < 0.01, 0.001, and not significant, respectively. GE = Gelatin, PE1 = Pea 1, PE2 = Pea 2, PT1 = Potato 1, PT2 = Potato 2, L = low dose, H = high dose, *(nr)* = no reduction occurred.

The first aim of red wine fining is to deplete an unbalanced astringency, which may be given from an excess of flavanols. However, a strong color loss affects negatively the wine quality. Therefore, an important key to the fining treatment is the selective reduction of unbalanced polymeric flavanol content combined with the lowest anthocyanin loss and, as a consequence, the highest final color intensity. In Figure 1, the polymeric flavanol (PRO) reduction was compared to the anthocyanin (TA) loss and to the color intensity (CI) decrease, expressed as a percentage.

Gelatin treatments were in general efficient in removing polymeric flavanols, with a dose-dependent reduction not only for PRO but also for CI in all the varieties. As well, TA removal was related to the dose in Montepulciano, whereas a higher gelatin dose was not corresponded by a higher TA decrease in Nebbiolo and Primitivo wines. Interestingly, in Syrah treated wines, the TA decrease was not significantly different for any of the investigated protein fining agents, in agreement with TA contents (Table 2), although CI variations were observed.

Pea-based proteins had a different effect depending on the formulation, the dose, and the variety evaluated (Figure 1). PE1 formulation gave interesting results for PRO removal in Primitivo and Nebbiolo wines. When PE1 was applied at a high dose, the PRO removal was similar to gelatin (GEH), even though differences were observed on the anthocyanin loss. In Primitivo wines, PE1H and GEH treatments caused similar TA reduction (−7.7% and −7.0% for PE1H and GEH, respectively), whereas in Nebbiolo the removed quantity of anthocyanins by PE1H was significantly lower than that reduced by gelatin treatments (−8.9% and −18.1%, corresponding to 29.3 mg and 36.7 mg of TA removed/g of PRO removed, for PE1H and GEH, respectively). On the other hand, the PRO removal by PE1 treatment at low dose was not significantly different to that corresponding to the high dose in Primitivo (−7.1% and −8.8% of PRO for PE1L and PE1H, respectively) but preserving anthocyanins, whereas in Nebbiolo wines similar removal of PRO and TA was obtained by using both low and high dose (27.3 mg and 29.3 mg of TA removed/g of PRO removed for PE1L and PE1H, respectively). In any case, a reduced loss of CI was observed for PE1L and PE1H treatments with respect to GEH in Nebbiolo and Primitivo wines. For PE1 formulation, the dose-response effect was negative on PRO removal for Syrah, namely the higher dose gave poorer results than the lower dose (−7.4% and −4.7% for PRO, resulting in 26.9 mg and 77.3 mg of TA removed/g of PRO removed for PE1L and PE1H, respectively). Regarding CI reduction, no significant difference was evidenced for all pea-based treatments in Syrah wines. The PE2H treatment was interesting in Syrah because it removed PRO similarly to gelatin (GEH) but preserving anthocyanins (−11.7% and −12.6% of PRO for PE2H and GEH, respectively). However, in Nebbiolo, the same treatment showed both low PRO removal (−7.9% for PE2H) and the highest removed amount of TA (−21.4% for PE2H, corresponding to 84.6 mg of TA removed/g of PRO removed), although there was not the same remarkable effect on the color intensity when compared to the other pea-based treatments.

Among potato-based treatments, high variability in the efficiency was found, particularly when applied to the different wines. Regarding the PRO removal, PT1 formulation at high dose gave similar results to gelatin (GEH) in Nebbiolo and Syrah wines, but anthocyanin losses and CI reduction were also similar (43.5 mg and 31.9 mg of TA removed/g of PRO removed for PT1H, 36.7 mg and 41.5 mg of TA removed/g of PRO removed for GEH, respectively, for Nebbiolo and Syrah). PT2H treatment also provided results in agreement with GEH for Syrah (42.6 mg of TA removed/g of PRO removed) with reduced CI decrease. In Nebbiolo wines, PT1L treatment reduced anthocyanin losses when compared to gelatin (−18.1%, −18.1%, and −8.0% for GEH, GEL, and PT1L, respectively) and therefore preserved better the wine color. In Primitivo and Montepulciano wines, also PT1L treatment gave the best results for potato-derived proteins, being worse the performance in relation to GEH and similar to GEL (–7.8% and –11.6% of PRO for PT1L; –10.3% and –12.0% of PRO for GEL, respectively, for Primitivo and Montepulciano wines). A non-significant dose-dependent reduction of PRO was observed for most of the potato-based formulations, but this relation was different depending on the variety evaluated. In fact, a smaller PRO removal was evidenced for PT1 at the high dose in Primitivo and Montepulciano, when compared to a low dose, whereas the PRO decrease was higher for high dose in Syrah and Nebbiolo wines.

In most cases, PT2 formulation showed the lowest effectiveness in PRO removal for all the varieties investigated, being it remarkably lower with respect to gelatin. Taking into account that the dose recommended by the supplier depends on the protein content, probably this formulation requires a much higher dose to appreciate its efficiency. In fact, only the highest recommended dose permitted the significant removal of oligomeric and polymeric flavanols in Syrah wine. However, it was evident that, even at low doses, anthocyanin contents were significantly reduced in Nebbiolo (up to −16.1%, *p* < 0.001, corresponding to a removal of 145 mg and 219 mg of TA/g of PRO for PT2L and PT2H, respectively) and a significant reduction of color intensity was also observed in Montepulciano, Syrah, and Nebbiolo with respect to control (Table 2). Therefore, this negative impact on wine color could be the limiting factor to an increased dosage of PT2.

Interestingly, the present study highlighted that the protein features, such as the proline content and the molecular mass [20,23], although of great relevance, are not the only ones influencing the effectiveness of red wine fining. In fact, earlier studies have evidenced that proline-rich proteins, such as pea-derived proteins, could interact more efficiently with galloylated flavanic derivatives [20]. In addition, molecular mass fractions ranging from 10 to 32 kDa act more specifically on astringent compounds, i.e., polymeric flavanols, whereas fractions between 30 and 42 kDa could be responsible for the pigment losses [23]. Therefore, the phenolic profile and concentration can play a key role in the hydrophobic interactions governing the insolubilisation and therefore the removal of target phenolic compounds involved in the perception of astringency and color. This was previously suggested by other authors but it had never been confirmed before.

**Figure 2:**
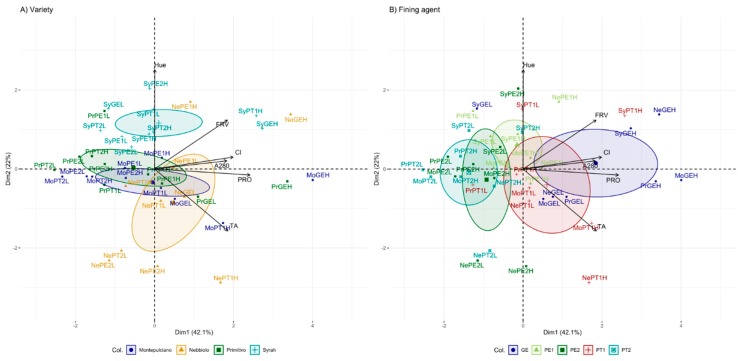
Principal component analysis (PCA) of phenolic compounds and color data obtained from wines after fining treatments with proteins of animal origin like gelatin (GE) and of vegetal origin from pea (PE1, PE2) and potato (PT1, PT2). PCA was performed on each parameter reduction with respect to the control. Confidence ellipses (*p* < 0.05) were drawn for variety (**A**) and fining agents formulation (**B**). Treatments corresponded to GE = Gelatin, PE1 = Pea 1, PE2 = Pea 2, PT1 = Potato 1, PT2 = Potato 2, L = low dose, H = high dose. Variety: Pr = Primitivo, Mo = Montepulciano, Sy = Syrah, Ne = Nebbiolo.

To better investigate the efficiency of protein treatments and the involved variables, a comparison of the different treatments was performed by PCA (Figure 2), with the aim of understanding if a general trend occurred for the same treatment (GE, PE1, PE2, PT1, and PT2), or if there was a stronger effect of the variety of features in the studied wines (Montepulciano, Primitivo, Nebbiolo, and Syrah). To minimize the variety differences, the multivariate comparison was done on the normalized data of each parameter reduction with respect to the control. Dimension 1 (D1) accounted for 42.1% of the explained variance, whereas dimension 2 (D2) explained 22.0% (total explained variance by the first two components = 64.1%). Dimension 1 was positively correlated, in order, with the decrease of PRO, CI, TA, FRV, and A_280_ (0.858, 0.706, 0.660, 0.656, and 0.651, respectively, all *p* < 0.001). Dimension 2 was strongly influenced by color parameters with a positive correlation with hue (0.893, *p* < 0.001) and a negative correlation with TA (−0.557, *p* < 0.001). As well, a less significant positive correlation was also found between D2 and FRV reduction (0.441, *p* < 0.001).

Regarding the variety effect (Figure 2A), variety features seem to have an important effect on the removal of phenolic compounds, being Syrah well separated from the other varieties, in particular from Nebbiolo, and D2 parameters (mainly hue loss) contributed strongly to the differentiation. Syrah wines, being rich in stable compounds from the chromatic point of view [30], fining treatments caused the shift to a red hue instead of yellowish (Table 2). Nebbiolo wines, having a higher ratio of di-substituted anthocyanin forms, are more prone to anthocyanin loss (Table 2) and consequently, the decrease of red color component (A_520_) was evidenced. Regarding vegetal fining agents, PT1 treatments (potato-derived protein, Figure 2B) were the most effective in the reduction of flavanol compounds, followed by PE1 (pea-based protein). PT1 treatments removed flavanols in a relatively similar way to gelatin whereas PE1 formulation preserved better-colored pigments. An important aspect to consider is the strong influence of formulation for the potato-based fining agents (PT1 and PT2) because PT2 treatment caused only a minor removal of flavanols and few changes in color parameters. Therefore, great variability in potato-based fining agents on the market can be assumed because of different sources, extraction conditions, extract preparation, and possible protein hydrolysis [1]. Undoubtedly, this leads to a different binding affinity and efficiency [23].

### 2.4. Astringency and Visual Color Assessment

The effect of the fining treatment on wine astringency was attempted by means of chemical and sensory methods in order to investigate if the significant reduction in the content of phenolic compounds effectively influenced astringency. The results obtained from the BSA precipitation index, as described by Boulet et al. [32], and from tasting sessions by a trained panel on astringency are shown in Table 3. Astringency determinations gave different results depending on the analytical method: the BSA index highlighted significant differences whereas the sensory-perceived intensity by the trained panel had not shown relevant differences in terms of astringency among fining treatments. For Syrah wine, a significant reduction in the BSA index was observed for gelatin at both low and high dose (–26.7%, *p* = 0.006 and –27.4%, *p* = 0.005 for GEL and GEH, respectively), and for PT1, PE1, and PE2 at high dose (–35.0%, *p* < 0.001, –25.2%, *p* = 0.010, and –25.8%, *p* = 0.009 for PT1H, PE1H, and PE2H, respectively) with respect to control wine. The highest decrease in the BSA index may be due to the greatest removal of polymeric flavanols (PRO, Table 1). Regarding Primitivo wine, a significantly lower BSA index was found after PT1H fining treatment (–34.6%, *p* < 0.001) when compared to control samples, although no reduction of A_280_, PRO, and FRV was reported (Table 1). As well, in Montepulciano and Nebbiolo wines, GEH and PT1H treatments also gave the lowest value of the BSA index, even though it is not significantly different from the control samples. In this case, the reductions were in accordance with a high decrease of FRV and PRO contents in Montepulciano, and of A_280_ and PRO in Nebbiolo (Table 1). Furthermore, in both Montepulciano and Nebbiolo wines, the trained panel perceived a lower intensity of astringency in the wines treated with PT1H fining agent with respect to the control, although the differences were not significant. The results obtained for PT1H formulation are in agreement with the effectiveness reported for potato protein in diminishing astringency of Aglianico wine [6].

The removal of flavanols, oligomeric or polymeric, is not always directly related to a reduction of astringency, either sensory perceived or chemically assessed. This could be due to the complexity of astringency sensation, which can be mainly triggered by polymeric flavanols of different subunit composition and modified by wine features such as alcohol content, acidity, and pH [32,33]. It is well known that the perceived astringency is increased at lower pH values. Furthermore, Quijada-Morín et al. [34] highlighted that polysaccharides, mainly mannoproteins and rhamnogalacturonan-II (RG-II), reduce astringency perception whereas the presence of mannose and galactose residues in the oligosaccharide fraction increases the perceived astringency.

**Figure 3:**
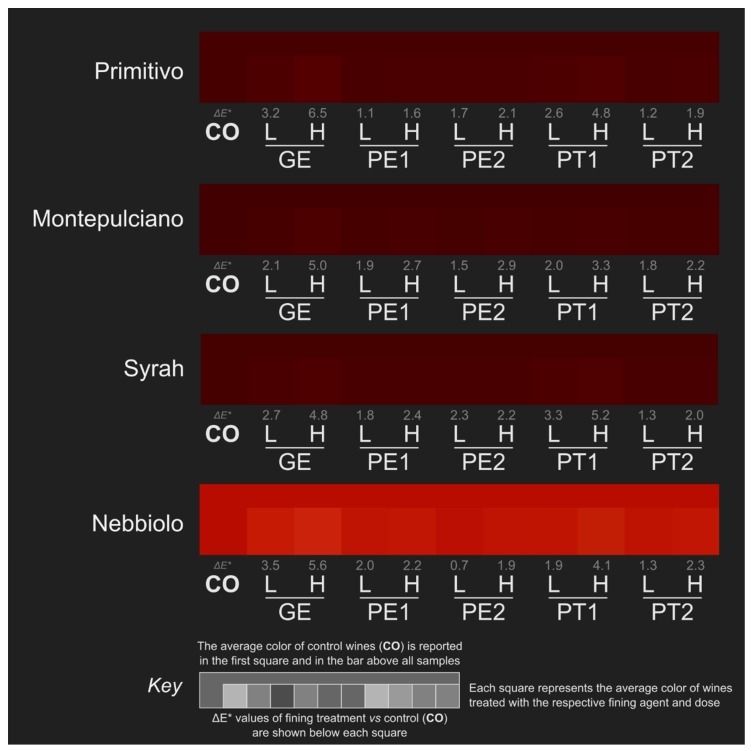
Wine color detected at the end of the treatment. Each wine color was acquired by spectrophotometry, expressed in CIE L*a* b* coordinates, and then converted to 8-bit RGB values for journal compatibility purposes. For each variety, the CO (Control) sample was extended on the left and top sides of the color bar to facilitate comparisons with treated wines in terms of colors. Treatments corresponded to GE = Gelatin, PE1 = Pea 1, PE2 = Pea 2, PT1 = Potato 1, PT2 = Potato 2, L = low dose, H = high dose.

Regarding visual color assessment, CIE L*a*b* coordinates (Table S1) were used to calculate the corresponding color on the RGB scale and the results are reported in Figure 3 for control and treated wines. In agreement with the significant changes observed in color intensity (Table 2), CIE L*a*b* parameters were strongly affected by fining treatments (L*, b*, *p* < 0.001 for all the varieties; a*, *p* < 0.01 for Nebbiolo and *p* < 0.001 for the other varieties, Table S1). This was particularly evident for GEH and PT1H treatments in Figure 3 for all the varieties evaluated. Lightness (L*) increased in the treated wines with respect to control samples, even though in different extent depending on the variety, the fining agent used, and the dose applied. The a* and b* coordinates (red/green color and yellow/blue color, respectively) also increased for Primitivo, Montepulciano, and Syrah wines, indicating a displacement towards reddish and yellowish hue, respectively. In contrast, b* values decreased in Nebbiolo wines treated with gelatin, PE1L, and PT1H showing a hue shifting to blue whereas a significant reduction in a*values were only found in PT1H treatment, thus reducing the red color component. In addition, CIE L*a*b* parameters highlighted the variety-dependent effect of the fining treatments on color modification. The three CIE L*a*b* coordinates increased significantly with GEL, GEH, and PT1H treatments in Primitivo wines, with all formulations at the high dose in Montepulciano, and with all fining treatments with the exception of PT2L in Syrah. In Nebbiolo wines, the three color coordinates were differently affected by the fining treatments.

To quantify the differences found in the wine color at the end of each fining treatment, ΔE* values were calculated with respect to control. This parameter was previously used to underline the ability of tasters to detect even small color differences in wines. While differences of one unit in this parameter could be detectable by tasters when the wine is directly observed [35], the ΔE* threshold suggested to correctly detect a wine color difference by the human eye was about 3 [36] or 5 units [37] probably as a consequence of color observation through a glass.

As can be observed in Figure 3, ΔE* data evidenced several important trends among fining agents for all the varieties evaluated. Gelatin treatments at high dose (GEH) gave the highest ΔE* values of the study, the resulting wines consistently achieving ΔE* values above 4.8 units. Only PT1 (potato-based) fining agent also at the high dose was found to cause a similar color reduction with ΔE* ranging from 3.3 to 5.2; these values being higher than those for GEH only in the case of Syrah wines. Furthermore, Primitivo and Nebbiolo wines treated with gelatin at a low dose (GEL) showed a visually perceived color reduction, as well for PT1 at a low dose (PT1L) in Syrah wine (ΔE* values between 3.2 and 3.5). No other pea and potato-based fining agent reached ΔE* values above three units, regardless of the dose considered.

The results obtained are consistent with previous studies on the impact on young wine color of fining treatments using gelatin and vegetal proteins from gluten [38]. In the cited study, darker colors were found in control wines, although lightness (L*) was not always significantly affected by the gelatin or gluten protein treatments.

## 3. Materials and Methods

### 3.1. Reagents and Standards

Chemicals of analytical reagent grade, cyanidin chloride, (+)-catechin, and bovine serum albumin were supplied by Sigma-Aldrich (St. Louis, MO, USA). The standard of malvidin-3-*O*-glucoside chloride was acquired from Extrasynthese (Genay, France). The solutions were prepared in deionized water produced by a Milli-Q system (Merck Millipore, Darmstadt, Germany).

### 3.2. Wines

Four young red wines (one wine for each variety, all belonging to vintage 2016) from Italian wineries were used in the present study for the fining trials: Primitivo, Montepulciano, Syrah, and Nebbiolo. They were selected on the basis of their different ratios between oligomeric and polymeric flavanols because of the impact on bitterness and astringency perception. The chemical characteristics of these four wines are shown in Table S2.

### 3.3. Wine Fining Trials

The fining agents used in this study were four commercially available and allergen-free vegetal-derived protein extracts; two from pea (PE1 and PE2) and other two from potato (PT1 and PT2). The study also included one animal gelatin (GE). For each fining agent, a stock solution of 10% *w*/*v* was prepared in deionized water with the exception of PT2, for which a stock solution of 2.5% *w*/*v* was done. Wine fining trials were carried out in completely filled 1-L bottles where two different dosages (Low and High) were added for each fining agent to each wine. Low (L) corresponds to the minimum dose +20% (i.e., +1/5) of the recommended dosage range whereas high (H) is the maximum dose –20% (i.e., –1/5) of the recommended dosage range (Table 4), representing doses commonly used for wine fining in industrial winemaking. For each wine, the control (CO) was prepared by adding deionized water instead of the fining agent.

All fining experiments were carried out in triplicate, by setting for 7 d at 18 °C. At the end of the fining treatment, wine samples were filtered on 40 μm cellulose filters (VWR International SAS, Leuven, Belgium). For each replicate, a 50 mL-aliquot of filtered wine was further centrifuged in a PK 131 centrifuge (ALC International, Milan, Italy) for 15 min at 3000× *g* at 20 °C and then used for chemical analysis. The remaining wine from each of the three replicates was homogenized, bottled, stored at 18 °C, and then used for sensory analysis.

### 3.4. Chemical Analysis after Wine Fining

Wine phenolic composition was determined through spectrophotometric methods using a UV-1800 spectrophotometer (Shimazdu Corp., Kyoto, Japan). In particular, total phenolics were assessed by the measurement of absorbance at 280 nm in deionized water (A_280_). Proanthocyanidins (PRO) were determined after acid hydrolysis at 100 °C using a ferrous salt (FeSO_4_) as catalyst according to the Bate-Smith reaction and expressed as mg of cyanidin chloride/L of wine. Monomeric and oligomeric forms of flavanols were evaluated as flavanols reactive to vanillin (FRV) and expressed as mg of (+)-catechin/L of wine. Total anthocyanins were determined by measuring absorbance at 536–540 nm after dilution with a hydroalcoholic solution composed of ethanol: water: 37% hydrochloric acid (70:30:1, *v*/*v*) and expressed as mg of malvidin-3-glucoside chloride/L of wine [39].

Flavanols contributing to astringency perception were evaluated following the Adams–Harbertson protein precipitation assay [40], modified by Boulet et al. [32]. Briefly, the wine was diluted with a buffer solution adjusted to pH 3.2, containing 12% *v*/*v* of ethanol and 5 g/L of tartaric acid. Then, 0.5 mL of diluted wine was added either with 1 mL of a buffer solution at pH 4.9 composed by 200 mM of acetic acid and 170 mM of NaCl or with 1 mL of the same buffer solution at pH 4.9 containing also 1 mg/mL of bovine serum albumin (BSA). After incubation at room temperature for 15 min with continuous agitation, the sample was centrifuged at 13,500× *g* for 5 min. The supernatants were diluted with 2% of hydrochloric acid and absorbance was measured at 280 nm. The BSA index was calculated as the difference between the two absorbance values and expressed as mg of (+)-catechin/L of wine.

### 3.5. Color Characteristics after Wine Fining

After the acquisition of visible spectra of undiluted samples using 1-mm optical path cuvettes, color intensity was calculated as the sum of absorbance measured at 420, 520, and 620 nm (A_420_ + A_520_ + A_620_ on an optical path of 10 mm) and hue was obtained as the ratio of absorbances measured at 420 and 520 nm (A_420_/A_520_) following the method OIV-MA-AS2-07B [41]. The wine color was also evaluated by the CIE L*a*b* parameters, namely lightness (L*), red/green color coordinate (a*), and yellow/blue color coordinate (b*), according to the method OIV-MA-AS2-11. The total color difference (ΔE*) between two samples (for example between a fined wine and the respective control wine) was calculated using the following expression: ΔE* = [(ΔL*)^2^ + (Δa*)^2^ + (Δb*)^2^]^1/2^ [41]. A UV-1800 spectrophotometer (Shimazdu Corporation) was used.

### 3.6. Sensory Analysis after Wine Fining

Sensory analysis was conducted by nine tasters (6 males and 3 females) aged between 30 and 55 years from the University of Turin researchers and professors with experience in wine sensory analysis. Panelists were trained during twelve sessions (1 h per session, three times per week) to recognize, standardize, and rate the perceived intensity of astringency. During the training sessions, panelists were asked to mark when they began to feel some stimuli and to order the samples on the basis of the perceived intensity. Triangle, duo-trio, and intensity tests on structured (1–10) and unstructured (10 cm) scales were performed using enological tannin (0.1–1 g/L) dissolved in water and wine.

Wine samples were then evaluated in five formal sessions to identify and rate the astringency perception. In the first session, the control wines were assessed to establish the astringency intensity of each non-treated wine. Then, all wines (control and fined) from the same variety were arranged in one session of 1 h, randomly presented to avoid a likely effect of context stimuli on ratings, and identified with three-digit random codes. A constant volume of 30 mL for each wine was evaluated in wine-taster glasses at 12 °C as described by the International Organization for Standardization (ISO) 3591 Norm (1977) [42]. Panelists recorded their perceived intensity on a 10-cm unstructured linear scale. To minimize carryover effects, panelists eat crackers between samples and then rinsed the mouth with natural water.

### 3.7. Statistical Analysis

Statistical analysis was conducted using R statistic software (R Core Team [43]). For each studied variable, one-way analysis of variance (ANOVA) using the Tukey HSD post-hoc test was used to evaluate significant differences among treatments. Levene’s and Shapiro–Wilk’s tests were applied for assessing the homogeneity of variance and normality of ANOVA residuals, respectively. Moreover, multiple comparison Dunnett’s test was performed to investigate significant differences between each fined wine and the respective control. Principal component analysis (PCA) using the R package “factoextra” [44] was performed on the ANOVA significant chemical parameters determined (*p* < 0.05) and therefore A_280_, PRO, FRV, TA, color intensity, and hue were the variables. To compare the effect of fining agents in varieties with different phenolic composition while minimizing the contribution of different phenolic content and profile, each value of the chemical parameters in fined wines was subtracted to that of the respective control wine and the difference was then normalized as *z*-scores before multivariate analysis.

## 4. Conclusions

Gelatin is the most commonly used fining agent in wine production, and results showed its ability and efficiency in the reduction of wine flavanol components, counter parted by a loss of anthocyanins to a different extent depending on the studied variety characteristics and the treatment dose. Plant-derived protein finings have been proposed to be a possible alternative. In particular, those derived from potato and pea could be useful for vegan-friendly, allergen-free wine production. The study tested the performance of several plant-derived agents on the red wine fining compared to gelatin.

In this study, fining agents derived from plants gave different results on phenolic compounds reduction, depending on their origin, formulation, dose applied, and also on the studied wine characteristics in terms of phenolic content and profile. Indeed, using four different monovarietal red wines exhibiting different phenolic features allowed us to highlight the effect of the wine composition, particularly on the phenolic characteristics, on the effectiveness of the fining treatment. Furthermore, the results suggest the necessity of preliminary trials for wine fining, possibly accompanied by instrumental measurements in terms of flavanols, astringency, and color.

## Figures and Tables

**Table 1:** Phenolic composition of red wines untreated (CO) and treated with fining agents of animal origin (GE) and vegetal origin from pea (PE1, PE2) and potato (PT1, PT2).

	Primitivo			Montepulciano			Syrah			Nebbiolo		
Treatment	A_280_	PRO	FRV	A_280_	PRO	FRV	A_280_	PRO	FRV	A_280_	PRO	FRV
	(AU, OP 10 mm)	(mg/L)	(mg/L)	(AU, OP 10 mm)	(mg/L)	(mg/L)	(AU, OP 10 mm)	(mg/L)	(mg/L)	(AU, OP 10 mm)	(mg/L)	(mg/L)
CO	68.8 ± 2.1ab	3254 ± 83a	1323 ± 42abc	84.1 ± 1.1	3440 ± 40a	1330 ± 39a	72.5 ± 1.4a	3094 ± 19a	1659 ± 9abc	56.7 ± 0.5a	3589 ± 16a	2129 ± 33ab
GEL	65.1 ± 2.1bc	2919 ± 76cd	1318 ± 59abc	84.7 ± 0.6	3025 ± 33c	1236 ± 55bc	70.9 ± 1.4ab	2991 ± 43ab	1587 ± 35abcd	52.4 ± 0.4cd	3330 ± 81cd	2085 ± 26b
GEH	62.9 ± 1.0c	2801 ± 66d	1229 ± 4cd	82.1 ± 0.9	2788 ± 44d	1092 ± 10d	66.7 ± 1.3b	2704 ± 30de	1495 ± 39d	51.8 ± 1.1d	3038 ± 0f	1836 ± 45e
PE1L	68.2 ± 1.4abc	3022 ± 87bc	1176 ± 25d	83.2 ± 0.2	3209 ± 79abc	1237 ± 50bc	70.5 ± 1.3ab	2863 ± 75bcd	1649 ± 6a	53.9 ± 0.8bc	3243 ± 77de	1971 ± 49cd
PE1H	68.5 ± 2.1ab	2969 ± 30bcd	1235 ± 13bcd	83.4 ± 2.1	3134 ± 71abc	1186 ± 31c	69.0 ± 2.4ab	2947 ± 33ab	1591 ± 30abcd	53.8 ± 0.4bc	3240 ± 100de	1907 ± 33de
PE2L	67.3 ± 1.9abc	3172 ± 64ab	1347 ± 20ab	85.1 ± 1.4	3262 ± 80ab	1304 ± 3ab	70.0 ± 1.5ab	2910 ± 30abc	1621 ± 40abc	54.8 ± 0.5b	3380 ± 14bcd	2196 ± 36a
PE2H	69.0 ± 0.4ab	3234 ± 43a	1255 ± 9bcd	84.9 ± 1.0	3178 ± 49abc	1306 ± 17ab	69.7 ± 2.0ab	2732 ± 103cde	1544 ± 40bcd	53.7 ± 0.3bc	3306 ± 75cde	2190 ± 22a
PT1L	71.4 ± 2.0a	3000 ± 56bcd	1337 ± 82abc	85.2 ± 1.2	3041 ± 30c	1181 ± 14c	70.0 ± 1.9ab	2935 ± 95ab	1492 ± 39d	54.6 ± 0.5b	3284 ± 103cde	1970 ± 47cd
PT1H	65.5 ± 1.0bc	3159 ± 81ab	1380 ± 39a	82.4 ± 2.2	3112 ± 57bc	1160 ± 5cd	68.0 ± 1.9ab	2620 ± 82e	1527 ± 14cd	52.5 ± 0.5cd	3125 ± 68ef	2058 ± 14bc
PT2L	68.6 ± 2.5ab	3182 ± 20ab	1372 ± 32a	84.4 ± 0.3	3334 ± 14a	1367 ± 19a	69.9 ± 1.0ab	2988 ± 56ab	1632 ± 9ab	54.1 ± 0.3bc	3464 ± 68abc	2151 ± 8ab
PT2H	69.8 ± 2.3ab	3125 ± 30abc	1261 ± 5bcd	85.7 ± 0.5	3212 ± 44abc	1312 ± 24ab	70.8 ± 1.4ab	2885 ± 62bcd	1507 ± 50d	53.3 ± 0.9bcd	3536 ± 47ab	2060 ± 24bc
ANOVA	***	***	***	ns	***	***	*	***	***	***	***	***
Contrasts’ significance *p*-values respect to control												
CO vs. GEL	0.076	**0.000**	1.000	0.999	**0.000**	**0.006**	0.789	0.309	0.627	**0.000**	**0.001**	0.530
CO vs. GEH	**0.001**	**0.000**	**0.021**	0.290	**0.000**	**0.000**	**0.002**	**0.000**	**0.001**	**0.000**	**0.000**	**0.000**
CO vs. PE1L	1.000	**0.004**	**0.000**	0.939	0.201	**0.006**	0.610	**0.002**	0.991	**0.000**	**0.000**	**0.000**
CO vs. PE1H	1.000	**0.000**	0.193	0.983	**0.015**	**0.000**	0.095	0.064	0.727	**0.000**	**0.000**	**0.000**
CO vs. PE2L	0.937	0.928	0.799	0.945	0.711	0.873	0.369	**0.013**	1.000	**0.009**	**0.008**	0.134
CO vs. PE2H	1.000	1.000	0.189	0.983	0.074	0.914	0.263	**0.000**	**0.049**	**0.000**	**0.000**	0.205
CO vs. PT1L	0.268	**0.001**	1.000	0.874	**0.000**	**0.000**	0.376	**0.038**	**0.001**	**0.003**	**0.000**	**0.000**
CO vs. PT1H	0.154	0.807	0.101	0.458	**0.006**	**0.000**	**0.020**	**0.000**	**0.014**	**0.000**	**0.000**	0.100
CO vs. PT2L	1.000	0.682	0.197	1.000	1.000	0.590	0.335	0.280	1.000	**0.000**	0.199	0.974
CO vs. PT2H	0.973	0.359	0.296	0.592	0.220	0.985	0.765	**0.004**	**0.003**	**0.000**	0.933	0.116

All data are expressed as average value ± standard deviation (n = 3). Different Latin letters within the same column indicate significant differences among treatments according to Tukey test (*p* < 0.05). Sign: *, ***, and “ns” indicate significance at *p* < 0.05, 0.001, and not significant, respectively. Contrasts values in bold face are significantly different from the control according to Dunnett test (*p* < 0.05). A_280_ = Absorbance measured at 280 nm, PRO = Proanthocyanidins expressed as mg of cyanidin chloride/L of wine, FRV = Flavanols reactive to vanillin expressed as mg of (+)-catechin/L of wine. CO = Control, GE = Gelatin, PE1 = Pea 1, PE2 = Pea 2, PT1 = Potato 1, PT2 = Potato 2, L = low dose, H = high dose.

**Table 2:** Total anthocyanins and color parameters of red wines untreated (CO) and treated with fining agents of animal origin (GE) and vegetal origin from pea (PE1, PE2) and potato (PT1, PT2).

	Primitivo			Montepulciano			Syrah			Nebbiolo		
Treatment	TA	Color Intensity	Hue	TA	Color Intensity	Hue	TA	Color Intensity	Hue	TA	Color Intensity	Hue
	(mg/L)	(AU, OP 10 mm)		(mg/L)	(AU, OP 10 mm)		(mg/L)	(AU, OP 10 mm)		(mg/L)	(AU, OP 10 mm)	
CO	255 ± 1abc	13.97 ± 0.20a	0.708 ± 0.002ab	275 ± 4a	15.12 ± 0.06a	0.664 ± 0.002b	367 ± 11a	13.73 ± 0.14a	0.677 ± 0.001a	112 ± 4a	4.73 ± 0.06a	1.028 ± 0.005bc
GEL	238 ± 3d	13.02 ± 0.01bc	0.708 ± 0.001ab	265 ± 2c	14.22 ± 0.12de	0.669 ± 0.002ab	367 ± 15a	13.08 ± 0.01bc	0.673 ± 0.001bc	92 ± 3de	4.25 ± 0.03de	1.028 ± 0.003bc
GEH	238 ± 2cd	12.19 ± 0.10d	0.709 ± 0.001a	257 ± 2d	13.35 ± 0.08f	0.668 ± 0.001ab	351 ± 7a	12.50 ± 0.12d	0.672 ± 0.001bc	92 ± 2de	3.99 ± 0.04f	1.022 ± 0.000c
PE1L	261 ± 3a	13.68 ± 0.05a	0.707 ± 0.001ab	271 ± 2abc	14.69 ± 0.07bc	0.666 ± 0.001b	361 ± 11a	13.31 ± 0.23bc	0.672 ± 0.001bc	102 ± 3b	4.45 ± 0.09c	1.030 ± 0.005abc
PE1H	236 ± 2cd	13.55 ± 0.05a	0.706 ± 0.001b	267 ± 0bc	14.45 ± 0.07cd	0.665 ± 0.002b	355 ± 9a	13.20 ± 0.13bc	0.672 ± 0.002bc	102 ± 4bc	4.42 ± 0.03cd	1.022 ± 0.003c
PE2L	253 ± 2abcd	13.59 ± 0.12a	0.707 ± 0.001ab	270 ± 2abc	14.82 ± 0.08ab	0.665 ± 0.001b	360 ± 10a	13.20 ± 0.06bc	0.674 ± 0.001ab	96 ± 1bcd	4.64 ± 0.05ab	1.036 ± 0.003ab
PE2H	243 ± 5bcd	13.39 ± 0.02ab	0.706 ± 0.001b	265 ± 3c	14.45 ± 0.16cd	0.664 ± 0.001b	371 ± 6a	13.17 ± 0.08bc	0.672 ± 0.001bc	88 ± 2e	4.46 ± 0.06bc	1.034 ± 0.008abc
PT1L	248 ± 8abcd	13.18 ± 0.06ab	0.708 ± 0.001ab	267 ± 0bc	14.48 ± 0.11cd	0.669 ± 0.002ab	364 ± 15a	12.94 ± 0.10c	0.674 ± 0.002abc	103 ± 3b	4.46 ± 0.08bc	1.035 ± 0.004ab
PT1H	245 ± 2bcd	12.65 ± 0.04cd	0.707 ± 0.001ab	258 ± 2d	13.90 ± 0.24e	0.672 ± 0.005a	352 ± 6a	12.50 ± 0.11d	0.670 ± 0.002c	92 ± 2de	4.22 ± 0.11e	1.041 ± 0.005a
PT2L	255 ± 2ab	13.67 ± 0.04a	0.709 ± 0.000ab	275 ± 1a	14.71 ± 0.07bc	0.666 ± 0.002b	363 ± 6a	13.43 ± 0.11ab	0.673 ± 0.001bc	94 ± 1cde	4.54 ± 0.03bc	1.035 ± 0.005ab
PT2H	251 ± 5abcd	13.48 ± 0.10ab	0.708 ± 0.000ab	272 ± 1ab	14.57 ± 0.08bc	0.666 ± 0.001b	358 ± 8a	13.20 ± 0.23bc	0.674 ± 0.002ab	100 ± 4bc	4.41 ± 0.01cd	1.031 ± 0.000abc
ANOVA	***	***	*	***	***	**	ns	***	***	***	***	***
Contrasts’ significance *p*-values respect to control												
CO vs. GEL	**0.004**	**0.001**	1.000	**0.000**	**0.000**	0.081	1.000	**0.000**	**0.006**	**0.000**	**0.000**	1.000
CO vs. GEH	**0.037**	**0.000**	0.635	**0.000**	**0.000**	0.240	0.295	**0.000**	**0.005**	**0.000**	**0.000**	0.413
CO vs. PE1L	0.081	1.000	0.606	0.122	**0.001**	0.763	0.980	**0.008**	**0.002**	**0.004**	**0.000**	0.999
CO vs. PE1H	**0.014**	0.997	0.189	**0.002**	**0.000**	0.999	0.671	**0.001**	**0.001**	**0.002**	**0.000**	0.342
CO vs. PE2L	1.000	1.000	0.395	**0.033**	**0.028**	0.999	0.949	**0.001**	0.128	**0.000**	0.435	0.271
CO vs. PE2H	0.339	0.405	0.251	**0.000**	**0.000**	1.000	0.998	**0.000**	**0.002**	**0.000**	**0.000**	0.612
CO vs. PT1L	0.843	**0.013**	1.000	**0.002**	**0.000**	**0.048**	1.000	**0.000**	0.093	**0.006**	**0.000**	0.322
CO vs. PT1H	0.373	**0.000**	0.635	**0.000**	**0.000**	**0.000**	0.364	**0.000**	**0.000**	**0.000**	**0.000**	**0.011**
CO vs. PT2L	0.939	1.000	0.999	1.000	**0.002**	0.912	1.000	0.073	**0.010**	**0.000**	**0.012**	0.413
CO vs. PT2H	1.000	0.852	0.996	0.670	**0.000**	0.827	0.864	**0.001**	0.128	**0.000**	**0.000**	0.996

All data are expressed as average value ± standard deviation (n = 3). Different Latin letters within the same column indicate significant differences among treatments according to Tukey test (*p* < 0.05). Sign: *, **, ***, and “ns” indicate significance at *p* < 0.05, 0.01, 0.001, and not significant, respectively. Contrasts values in bold face are significantly different from the control according to Dunnett test (*p* < 0.05). TA = Total anthocyanins expressed as mg of malvidin-3-glucoside chloride/L of wine. CO = Control, GE = Gelatin, PE1 = Pea 1, PE2 = Pea 2, PT1 = Potato 1, PT2 = Potato 2, L = low dose, H = high dose.

**Table 3:** Astringency determined by chemical and sensory analysis of red wines untreated (CO) and treated with fining agents of animal origin (GE) and vegetal origin from pea (PE1, PE2) and potato (PT1, PT2).

Treatment	Primitivo		Montepulciano		Syrah		Nebbiolo	
	Sensory	BSA Index (mg/L catechin)	Sensory	BSA Index (mg/L catechin)	Sensory	BSA Index (mg/L catechin)	Sensory	BSA Index (mg/L catechin)
CO	6.54 ± 0.47	1008 ± 113abc	5.43 ± 0.33	601 ± 74abc	4.75 ± 0.67	889 ± 68a	7.54 ± 0.43	585 ± 67ab
GEL	5.25 ± 0.65	957 ± 59bc	4.70 ± 0.84	431 ± 47c	3.69 ± 1.02	652 ± 85b	6.16 ± 0.39	723 ± 49a
GEH	4.89 ± 0.47	912 ± 28c	4.69 ± 0.43	539 ± 62abc	3.51 ± 0.79	646 ± 85b	7.06 ± 0.44	527 ± 27b
PE1L	4.79 ± 0.61	1189 ± 83ab	4.54 ± 0.67	744 ± 87a	4.50 ± 0.80	681 ± 63ab	6.47 ± 0.53	631 ± 68ab
PE1H	4.73 ± 0.86	1057 ± 151abc	4.37 ± 0.60	467 ± 111bc	4.38 ± 1.02	665 ± 105b	6.63 ± 0.79	594 ± 15ab
PE2L	3.70 ± 0.63	1035 ± 144abc	3.94 ± 0.65	477 ± 61bc	3.84 ± 0.95	712 ± 33ab	6.59 ± 0.45	585 ± 34ab
PE2H	4.35 ± 0.70	1214 ± 27a	4.52 ± 0.80	494 ± 44abc	4.24 ± 0.80	660 ± 79b	5.60 ± 0.82	586 ± 86ab
PT1L	6.04 ± 0.46	889 ± 96cd	4.20 ± 0.62	630 ± 74abc	4.33 ± 0.69	675 ± 75ab	6.57 ± 0.42	615 ± 20ab
PT1H	5.39 ± 0.68	660 ± 92d	3.41 ± 0.64	417 ± 102c	4.19 ± 0.96	578 ± 76b	6.02 ± 0.38	570 ± 24b
PT2L	5.04 ± 0.73	968 ± 46bc	4.26 ± 0.76	718 ± 77ab	3.38 ± 1.13	693 ± 50ab	6.68 ± 0.65	597 ± 25ab
PT2H	4.95 ± 0.49	835 ± 98cd	3.60 ± 0.64	646 ± 182abc	3.66 ± 0.90	760 ± 95ab	6.30 ± 0.38	573 ± 69b
ANOVA	ns	***	ns	**	ns	**	ns	*
*Contrasts’ significance p-values respect to control*								
CO vs. GEL	0.637	0.981	0.981	0.193	0.972	**0.006**	0.375	**0.019**
CO vs. GEH	0.352	0.640	0.978	0.972	0.932	**0.005**	0.996	0.663
CO vs. PE1L	0.289	**0.075**	0.937	0.344	1.000	**0.019**	0.666	0.843
CO vs. PE1H	0.254	0.984	0.843	0.418	1.000	**0.010**	0.820	1.000
CO vs. PE2L	**0.015**	1.000	0.515	0.500	0.990	**0.056**	0.781	1.000
CO vs. PE2H	0.105	**0.035**	0.928	0.663	1.000	**0.009**	**0.086**	1.000
CO vs. PT1L	0.998	0.413	0.721	1.000	1.000	**0.015**	0.761	0.984
CO vs. PT1H	0.751	**0.000**	0.189	0.135	1.000	**0.000**	0.277	1.000
CO vs. PT2L	0.461	1.000	0.764	0.577	0.884	**0.029**	0.855	1.000
CO vs. PT2H	0.396	**0.097**	0.280	0.996	0.968	0.256	0.503	1.000

Sensory data are expressed as average value ± error calculated as *sd/(n)^1/2^*, where *sd* is the standard deviation and *n* is the number of panelists (*n* = 9). BSA data are expressed as average value ± standard deviation (*n* = 3). Different Latin letters within the same column indicate significant differences among treatments according to Tukey test (*p* < 0.05). Sign: *, **, ***, and “ns” indicate significance at *p* < 0.05, 0.01, 0.001, and not significant, respectively. Contrasts values in bold face are significantly different from the control according to Dunnett test (*p* < 0.1). CO = Control, GE = Gelatin, PE1 = Pea 1, PE2 = Pea 2, PT1 = Potato 1, PT2 = Potato 2, L = low dose, H = high dose.

**Table 4:** Fining agents used in this experiment.

Code	Origin	Recommended Dose (g/hL)	Low Dose (g/hL)	High Dose (g/hL)
CO	-	-	-	-
GE	Animal	5–30	10	25
PE1	Pea	10–20	12	18
PE2	Pea	5–20	8	17
PT1	Potato	5–30	10	25
PT2	Potato	2–5	2.6	4.4

## Supplementary Materials

The following are available online, chemical composition of initial wines and CIE L*a*b* coordinates of control and treated wine.
