# Supplementary material for: Phenolic Composition Influences the Effectiveness of Fining Agents in Vegan-Friendly Red Wine Production
Source: Molecules. 2019 Dec 28;25(1):120. doi: 10.3390/molecules25010120 (PMC6983225; doi:10.3390/molecules25010120)
Supplement: Supplementary file 1 [file molecules-25-00120-s001.pdf]

Table S1. CIE L*a*b* coordinates for red wines untreated (CO) and treated with fining agents of animal origin (GE) and vegetal origin from pea (PE1, PE2) and potato (PT1, PT2).

Variety	Treatment	L*	a*	b*
Primitivo	CO	6.8 ± 0.2d	36.14 ± 0.42d	11.71 ± 0.35d
	GEL	8.0 ± 0.1bc	38.20 ± 0.09bc	13.81 ± 0.10bc
	GEH	9.4 ± 0.2a	40.04 ± 0.22a	16.16 ± 0.27a
	PE1L	7.2 ± 0.1d	36.94 ± 0.28cd	12.39 ± 0.25d
	PE1H	7.4 ± 0.1cd	37.25 ± 0.22cd	12.69 ± 0.20cd
	PE2L	7.4 ± 0.2cd	37.32 ± 0.32cd	12.74 ± 0.30cd
	PE2H	7.6 ± 0.1cd	37.56 ± 0.16cd	13.03 ± 0.15cd
	PT1L	7.8 ± 0.1cd	37.86 ± 0.23bcd	13.39 ± 0.24cd
	PT1H	8.7 ± 0.1ab	39.11 ± 0.09ab	14.94 ± 0.11ab
	PT2L	7.2 ± 0.1d	36.99 ± 0.20cd	12.44 ± 0.17d
	PT2H	7.5 ± 0.1cd	37.47 ± 0.18cd	12.92 ± 0.17cd
	Sign	***	***	***
Montepulciano	CO	6.2 ± 0.1c	34.48 ± 0.22c	10.66 ± 0.15c
	GEL	6.9 ± 0.2bc	36.02 ± 0.43b	11.95 ± 0.35bc
	GEH	8.1 ± 0.1a	37.81 ± 0.21a	13.87 ± 0.26a
	PE1L	6.8 ± 0.1bc	35.88 ± 0.21bc	11.72 ± 0.17bc
	PE1H	7.1 ± 0.2b	36.45 ± 0.32ab	12.22 ± 0.27b
	PE2L	6.7 ± 0.2bc	35.59 ± 0.37bc	11.47 ± 0.28bc
	PE2H	7.2 ± 0.3b	36.60 ± 0.50ab	12.34 ± 0.44b
	PT1L	6.9 ± 0.2bc	35.95 ± 0.45bc	11.83 ± 0.36bc
	PT1H	7.4 ± 0.7ab	36.71 ± 1.30ab	12.71 ± 1.23ab
	PT2L	6.8 ± 0.1bc	35.86 ± 0.24bc	11.71 ± 0.19bc
	PT2H	6.9 ± 0.1bc	36.14 ± 0.25b	11.96 ± 0.21bc
	Sign	***	***	***
Syrah	CO	6.5 ± 0.3d	35.37 ± 0.65d	11.26 ± 0.48d
	GEL	7.5 ± 0.0bc	37.26 ± 0.03bc	12.91 ± 0.02bc
	GEH	8.4 ± 0.2a	38.48 ± 0.31a	14.39 ± 0.36a
	PE1L	7.1 ± 0.3c	36.65 ± 0.51bc	12.31 ± 0.45c
	PE1H	7.4 ± 0.2bc	37.06 ± 0.39bc	12.68 ± 0.37bc
	PE2L	7.3 ± 0.1bc	37.03 ± 0.25bc	12.65 ± 0.22bc
	PE2H	7.3 ± 0.2bc	36.92 ± 0.33bc	12.58 ± 0.30bc
	PT1L	7.8 ± 0.1b	37.67 ± 0.13ab	13.35 ± 0.15b
	PT1H	8.5 ± 0.1a	38.76 ± 0.17a	14.66 ± 0.21a
	PT2L	7.0 ± 0.1cd	36.35 ± 0.19cd	12.04 ± 0.16cd
	PT2H	7.2 ± 0.4bc	36.76 ± 0.71bc	12.45 ± 0.66bc
	Sign	***	***	***
Nebbiolo	CO	33.5 ± 0.6f	53.11 ± 0.67a	45.72 ± 0.57a
	GEL	36.9 ± 0.3b	53.28 ± 0.37a	44.63 ± 0.19bc
	GEH	38.8 ± 0.2a	52.74 ± 0.19ab	43.74 ± 0.34cd
	PE1L	35.2 ± 0.8cde	52.79 ± 0.42ab	44.69 ± 0.23bc
	PE1H	35.7 ± 0.2bcd	53.22 ± 0.14a	45.24 ± 0.10ab
	PE2L	34.1 ± 0.4ef	52.70 ± 0.34ab	45.43 ± 0.18ab
	PE2H	35.2 ± 0.4cde	52.84 ± 0.51ab	44.92 ± 0.69ab
	PT1L	35.3 ± 0.6cde	52.90 ± 0.31a	45.29 ± 0.32ab
	PT1H	36.7 ± 1.0bc	51.61 ± 0.78b	43.49 ± 0.08d
	PT2L	34.8 ± 0.3def	53.03 ± 0.22a	45.30 ± 0.19ab
	PT2H	35.7 ± 0.1bcd	53.15 ± 0.31a	44.97 ± 0.29ab
	Sign	***	**	***

All data are expressed as average value ± standard deviation (n=3). Different Latin letters within the same column indicate significant differences among treatments according to Tukey test ($p < 0.05$). Sign: ** and *** indicate significance at $p < 0.01$ and 0.0001, respectively. L* = Lightness, a* = Red/green colour coordinate, b* = Yellow/blue colour coordinate. CO = Control, GE = Gelatin, PE1 = Pea 1, PE2 = Pea 2, PT1 = Potato 1, PT2 = Potato 2, L = low dose, H = high dose.

Table S2. Chemical composition of Primitivo, Montepulciano, Syrah, and Nebbiolo wines used for fining experiments.

Parameter	Primitivo	Montepulciano	Syrah	Nebbiolo
Ethanol (% v/v)	14.6	12.4	14.4	13.2
Residual sugars (g/L)	4.56	2.42	1.43	0.33
Titratable acidity (g/L as tartaric acid)	6.4	5.8	5.2	5.3
pH	3.64	3.50	3.65	3.59
Tartaric acid (g/L)	1.49	2.40	1.90	1.60
Malic acid (g/L)	nd	nd	nd	nd
Lactic acid (g/L)	1.44	1.06	1.45	2.31
Acetic acid (g/L)	0.63	0.59	0.51	0.51
Succinic acid (g/L)	0.79	0.78	0.77	1.03
Glycerol (g/L)	12.80	8.94	9.38	9.55

All data are expressed as average value (n=3). nd = not detected.
